# Role of medical Thoracoscopy in the Management of Multiloculated Empyema

**DOI:** 10.1186/s12890-018-0745-y

**Published:** 2018-11-29

**Authors:** Kamran Khan Sumalani, Nadeem Ahmed Rizvi, Asif Asghar

**Affiliations:** 1 Department of Chest Medicine, Jinnah Postgraduate Medical Centre, Karachi, 75400 Pakistan; 2 Department of Thoracic Surgery, Combined Military Hospital, Peshawar, Pakistan

**Keywords:** Thoracoscopy, Empyema pleural, Tuberculosis, Inflammation, Video-assisted thoracoscopic surgery

## Abstract

**Background:**

The treatment of early pleural empyema depends on the treatment of ongoing infection by antimicrobial therapy along with thoracocentesis. In complicated empyema this treatment does not work and lung will not expand until removal of adhesions. The objective of the current study is to analyze the experience of management of multiloculated, exudative and fibrinopurulent empyema through rigid medical thoracoscopy under local anaesthesia and to explore new ways to manage the entity.

**Methods:**

This is a descriptive case series in which 160 patients were recruited through non-probability convenient sampling, from department of pulmonology, Jinnah postgraduate medical centre, Karachi, from September 2014 to August 2016. All patients underwent medical thoracoscopy under local anesthesia. Written Informed consent was taken from the study participants. Ethical approval was obtained from Ethical Review Committee of the hospital. Patients age > 70 years, those with multiple organ failure and bleeding disorders were excluded.

**Results:**

Out of 160 patients, 108 (67.50%) were male and 52 (32.5%) were female with mean age 25.37 years (range 16 to 70 years). Out of total, 102 (63.7%) had tuberculous empyema, while pleural biopsy of 58 (36.3%) patients was suggestive of non-tuberculous empyema. Final evolution through chest x-ray revealed complete resolution in 92 (57.5%), partial resolution in 58 (36.25%) patients. 9 (5.6%) developed persistent air leak while 1 (0.6%) patient expired due to urosepsis.

**Conclusion:**

Medical Thoracoscopy under local anesthesia is a safe, efficient and cost effective intervention for management of complicated empyema, particularly in resource constraint settings.

## Background

Pleural empyema is defined as pus accumulation in the pleural space; it is associated with significant morbidity and mortality in adults and children [1]. It can be subdivided into three stages: Stage 1: exudative which is freely moving pleural fluid, Stage 2: fibrino-purulent in which fibrin deposits on the pleural surfaces with a turbid, viscous fluid which has a tendency to loculate, and Stage 3: organizing which is characterized by fibrous thickening of the visceral pleura leading ultimately to a trapped lung by fibrous adhesions on pleura. [2, 3].

Ultrasound chest outperforms CT scan chest in visualizing septations and loculations within empyema. Diaphragmatic movement can be visualized in real-time which is reduced in heavily septated effusions or fibrothorax [4].

The visualization of these radiological features is useful in practice as the therapeutic approach clearly differs even when clinical trials to guide treatment are still lacking [5]. However, medical thoracoscopy is a least invasive procedure that provides access to the pleural space using a combination of visualizing and working instruments.

The term thoracoscopy is confusing because it refers to both the medical and surgical procedures. To avoid confusion, some authors suggest that medical thoracoscopy should be referred to as pleuroscopy. The term thoracoscopy may be used exclusively for the surgical thoracoscopic procedure [6, 7]. Advances in medicine and medical instruments, thoracoscopy has now become a routine procedure in the management of the disease of the chest including pleura.

In empyema, medical thoracoscopy is a drainage procedure, midway between tube thoracostomy and video-assisted thoracoscopic surgery (VATS), which is efficient, significantly cheaper and prevents from surgical thoracoscopy under general anaesthesia. It is important that it is performed in the start of disease and is particularly advisable for frail patients at high risk of surgery [8].

**Fig. 1:**
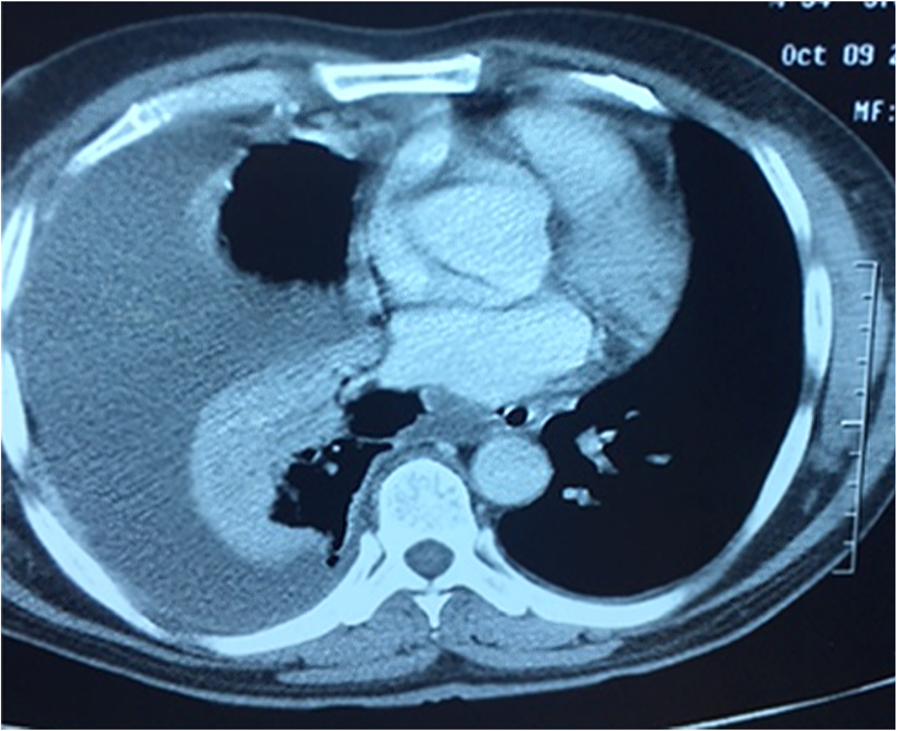
CT scan of the chest showing loculated empyema

No local data is available on this subject. Thus, we planned to conduct this study to report our experience about the effectiveness and safety of medical thoracoscopy in multiloculated, exudative and fibrinopurulent empyema confirmed by chest ultrasonography and CT scan. (Fig. 1).

## Methods

This was a case series in which 160 out of 199 patients with empyema were recruited from department of pulmonology, Jinnah Postgraduate Medical Centre, Karachi Pakistan from September 2014 to August 2016. This study was performed in accordance with the Declaration of Helsinki. This human study was approved by Ethical review committee Jinnah postgraduate medical center, Karachi. All adult participants provided written informed consent to participate in this study.

All patients underwent chest radiography and CT scan chest for confirmation of the disease. Ultrasonography of chest was done in all patients to localize the effusion, assess the echogenicity, diaphragmatic motility and presence of septations and loculations. For ultrasonographic chest examination convex transducer with frequency of 3.5–5 MHz was used and the examination was performed by a group of pulmonologists of the department who had received specific training and performed the examination on regular basis.

### Inclusion criteria

- Prolong presentation of empyema (> 30 days)
- No response to antibiotics therapy
- Failure of complete drainage by tube thoracostomy
- Multi-septated, multi-loculated empyema as evidenced by ultrasonography

### Exclusion criteria

- Age more than 70 years
- Consent not given for the procedure
- Bleeding disorders
- Multi-organ failure
- Hemodynamic instability
- Simple empyema, without septations and loculations
- Organized stage 3 empyema as evident on chest CT scan or ultrasound
- Terminally ill patients

Out of 199, thirty nine patients were excluded as 12 did not give consent for the procedure, 2 had acute myocardial infarction, 10 were hemodynamically unstable, and 11 patients had organized empyema. 4 patients did not meet the age criteria of the study so were excluded.

Thoracoscopy was performed in a dedicated endoscopic suite with facilities of ultrasonography. The procedure was fully informed to the patient. Prior to the procedure intercostal nerve block was given at paravertebral area in multiple adjacent intercostal spaces. Local anesthetic is administered with a syringe facing upwards at an angle of 20^o^ to approach the neurovascular bundle at the lower border of the rib. After confirmation by aspiration of blood, 3–5 mL of local anesthetic (1% solution of lidocaine, total 30 - 50 mg) is administered. The whole procedure is repeated for other levels of intercostal blockade needed.

Patient placed in a lateral decubitus position with empyema side up and arm above the head. Vitals and oxygen saturation were continuously monitored during the procedure. The incision site was anesthetized locally and adequate analgesia and conscious sedation was achieved so that patient can tolerate the procedure while maintaining cardiorespiratory functions. Lidocaine with adrenaline 3 mL of 1% solution (not exceeding 4.5 mg/kg) was used for local anesthesia. Drugs used for analgesia and sedation were nalbuphine in a dose of 0.3–3 mg/kg IV over 10–15 min, and midazolam 0.5–1 mg IV given over 2 min, not exceeding 2.5 mg/dose.

**Fig. 2:**
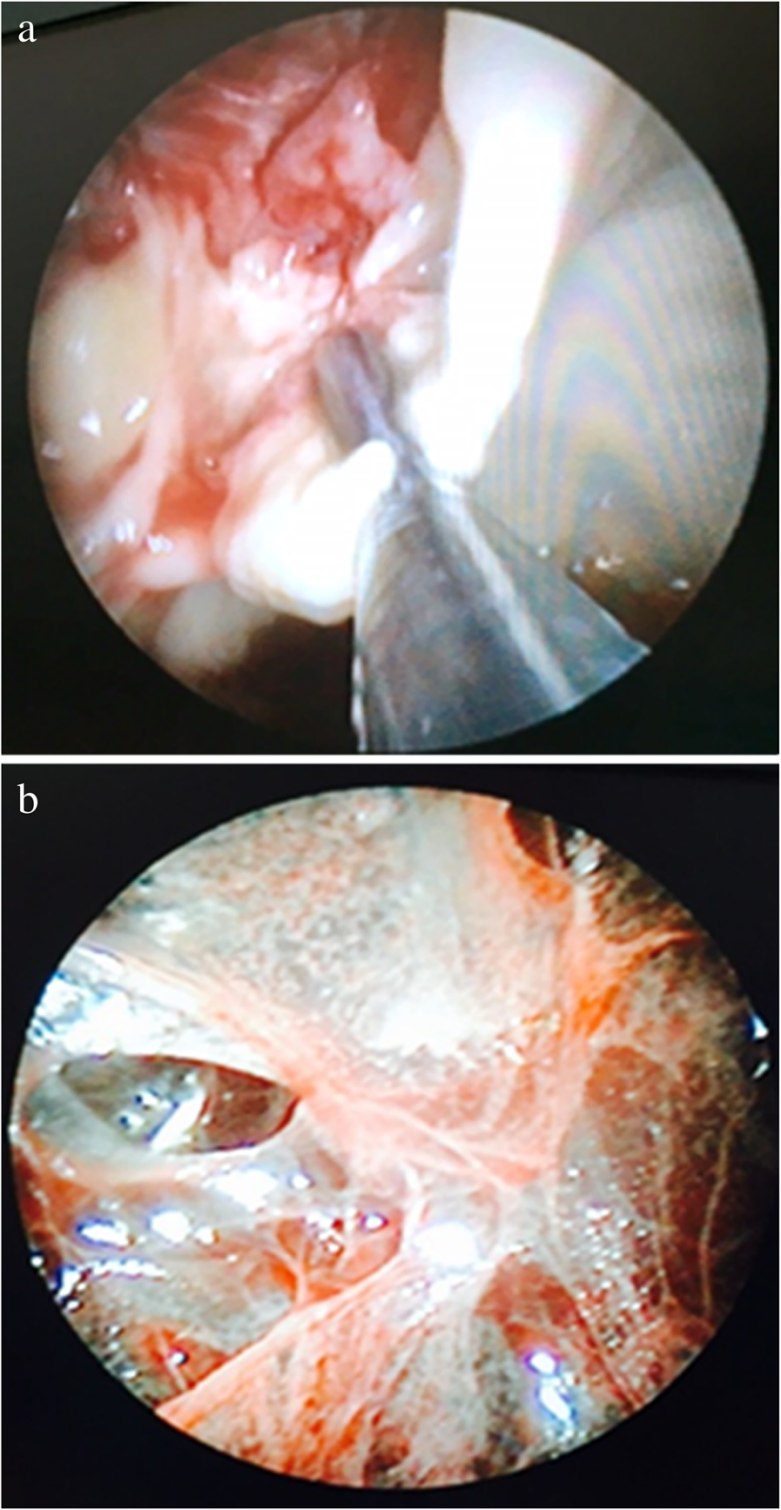
**a** Thick loculated empyema as seen on Thoracoscopy. Forceps introduced into pleural cavity to remove slough. **b** Fibrin membrane in pleural cavity as seen on thoracoscopy

Single port was made using 10 mm trocar under direct ultrasound guidance. Optical telescope at 0^o^ was inserted into the pleural cavity for video inspection. Fibrinous septa were removed with forceps, (Fig. 2a and b) while in case of thick debris and adhesions a second port was made to introduce large forceps.

Multiple targeted biopsies were also taken from the parietal pleura. At the end single or dual (28–32 Fr) chest tube(s) were placed in the cavity and attached to under-water seal with a suction pressure of − 20 cmH_2_O. Patients were encouraged to use incentive spirometer and early mobilization with the help of training stairs and bicycle ergometers.

All patients received broad spectrum 3rd generation cephalosporins after the procedure. Antibiotics were changed later on if needed on the basis of pleural fluid culture and sensitivity results. Patients diagnosed as having tuberculous empyema were treated with anti-tuberculous therapy. After successful procedure average time for chest tube removal was 7–25 days.

Complete resolution was labelled when opacity on chest radiograph was reduced to less than one-third of the hemithorax, and partial resolution was labelled when it was more than one-third of the hemithorax.

Treatment success included both complete and partial resolution cases. It was defined as radiologic confirmation of successful pleural drainage with no recurrence and hence no need for further treatment i.e. subsequent tube thoracostomies or surgical interventions and objective evidence of resolution of sepsis (improvement in temperature and clinical condition and decreasing inflammatory markers, total white cell count and serum C-reactive protein) at the time of discharge from hospital.

### Statistical analysis

Data was analysed on Statistical analysis software package (SPSS) version 21. Mean, frequencies and percentages were calculated for quantitative data. Chi-square test was used and *P* < 0.05 was considered significant with confidence interval taken as 95%.

## Results

**Fig. 3:**
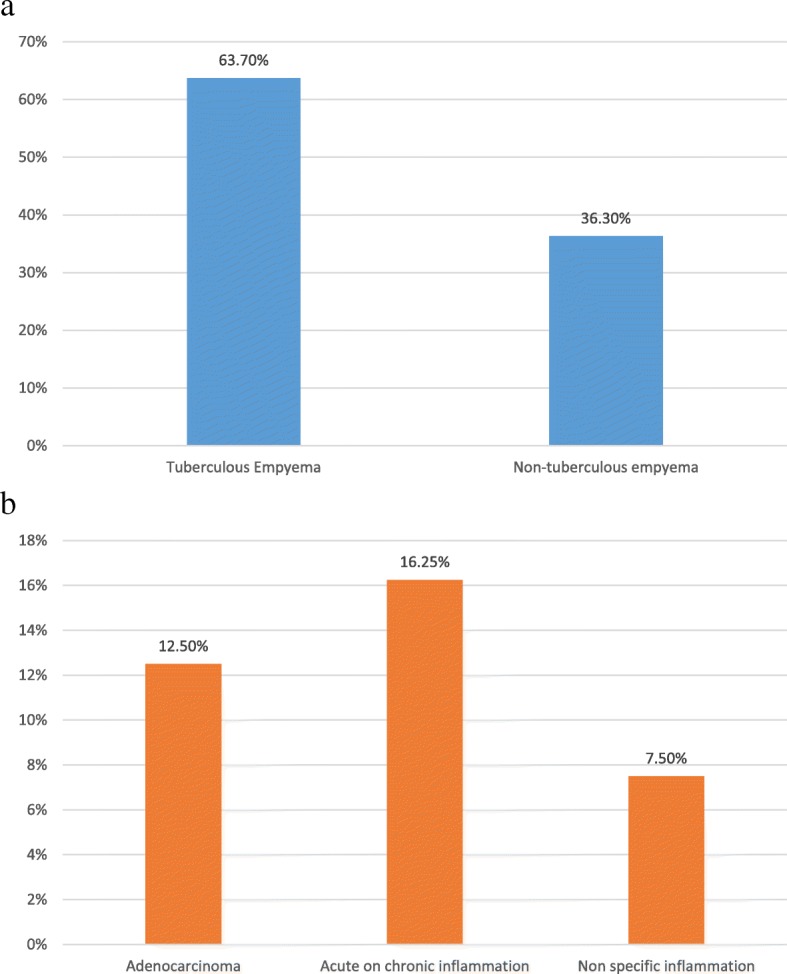
**a** Etiology of empyema as confirmed by thoracoscopic pleural biopsy **b**: Etiology of non-tuberculous empyema as confirmed by thoracoscopic pleural biopsy

A total of 160 patients with multi-loculated empyema were included in the study. Males were 108 (67.5%) and females 52 (32.5%) with the mean age of the study participants was 25.37 years (range: 19–60 years). The etiology of empyema as confirmed by thoracoscopic pleural biopsy as shown in table 1 and Fig. 3a and b.

**Table 1:** Etiology of empyema as confirmed by thoracoscopic pleural biopsy

Etiology	n (%)
Tuberculosis	102/160 (63.7)
Non-tuberculous	58/160 (36.36)
Acute on chronic inflammation	26/58 (16.25)
Malignancy(Adenocarcinoma)	20/58 (12.5)
Non-specific inflammation	12/58 (7.5)

n is the number of cases. Percentage is shown in parenthesis

**Fig. 4:**
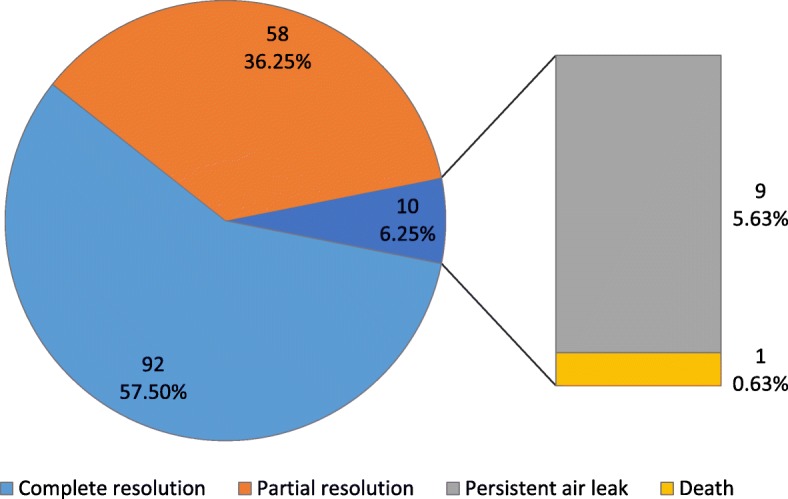
Outcome of radiograph resolution

As shown in Fig. 4, follow-up chest radiograph shows complete resolution in 92 (57.5%) and partial resolution in 58 (36.25%) patients with no further intervention.

**Fig. 5:**
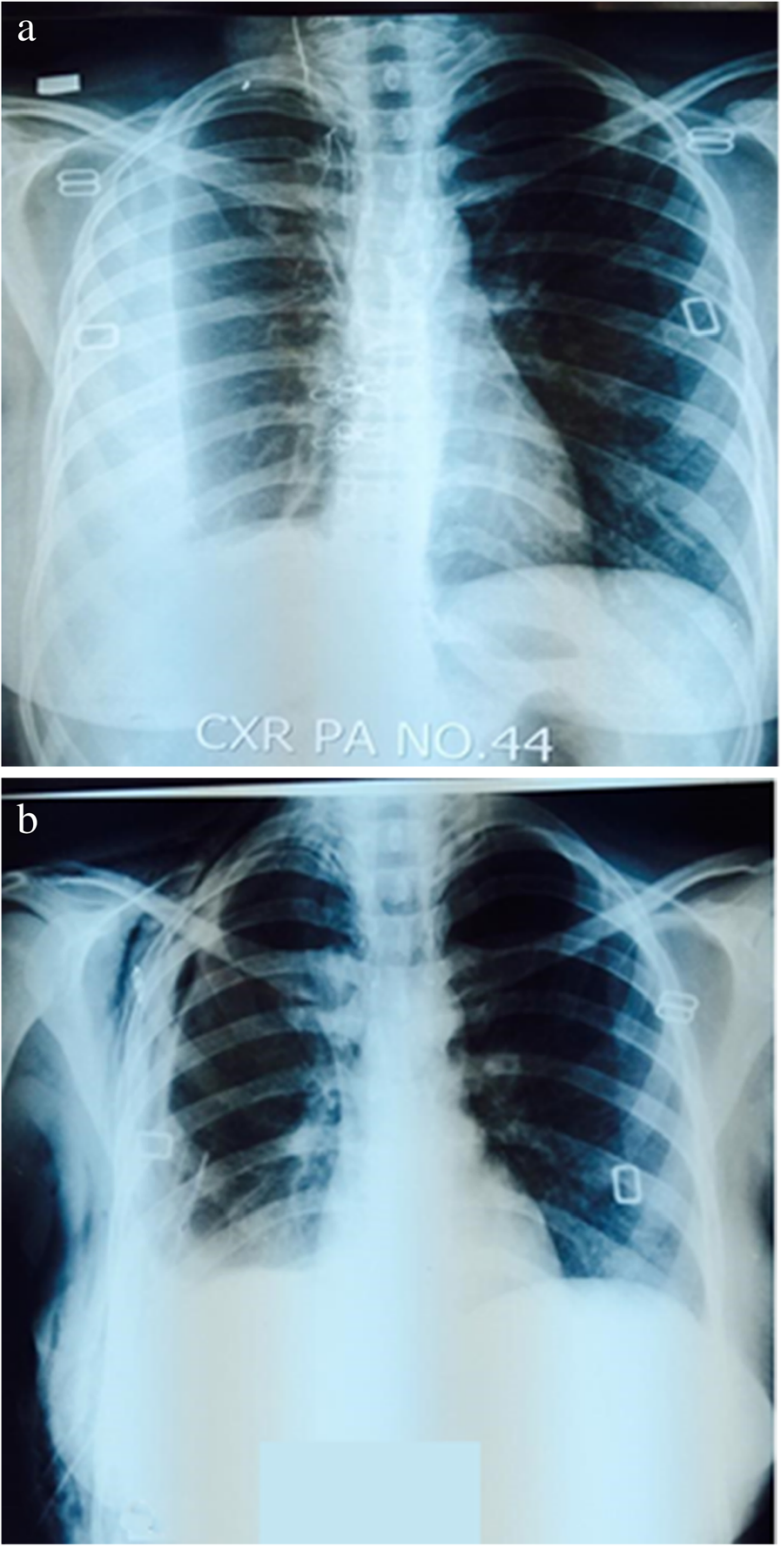
Comparison of chest X-rays before and after medical Thoracoscopy (**a**) chest x-ray before thoracoscopy showing loculated pleural effusion (**b**) chest x-ray after thoracoscopy showing complete resolution of multiloculated and organized empyema

Treatment success of 93.75% was achieved. A total of 2 complications were observed after the procedure; 9 (5.6%) of the patients developed persistent air leak and required surgical intervention and only one death was reported on 4th day post-procedure due to urosepsis. Comparison of chest X-rays before and after the procedure is shown in Fig. 5.

## Discussion

In our study we assessed the efficacy and safety of rigid medical thoracoscopy in cases of multi-loculated empyema stratified by chest ultrasonography. Treatment success rate with rigid medical thoracoscopy was found to be 93.75%. This included both complete and partial resolution cases. It was defined as radiologic confirmation of successful pleural drainage with no recurrence and hence no need for further treatment i.e. later tube thoracostomies or surgical procedures and sign of resolution of sepsis like decrement in total leukocyte count, serum C-reactive protein and fever at the time of discharge from hospital, i.e. after 7–25 days. The remaining 6.25% cases were labelled as treatment failure who required further surgical intervention.

Medical thoracoscopy is at the stage of its inception in Pakistan and there are only few centers scattered in the country doing this procedure, while the use of this minimally invasive procedure was introduced in 1910 in Europe to diagnose and treat pleural diseases [4, 9, 10].

The technique is similar to chest tube insertion with advantage that the pleural space can be visualized, biopsies can be taken, multiple loculations can be converted into a single communicating cavity and adequate drainage of fluid and thick fibrinous debris can be removed. The main advantage of medical thoracoscopy over VATS (video assisted thoracoscopy) is that it is less invasive, cheap procedure which can be done in an endoscopic suite and is better tolerated in frail patients who are under high risk with general anesthesia and tracheal intubation [11].

We did not use intrapleural fibrinolytic agents before or after the intervention in our cases due to high cost of some drugs and unavailability of others in the country. But as compared to failure rate (29%) of a large trial [11] using streptokinase and tube thoracostomy, the failure rate of medical thoracoscopy was only 6.25% in our study, 9 patients had persistent air leak which needed further surgical interventions and there was one death due to urosepsis. The most common cause of exudative pleural effusion/empyema in Pakistan is tuberculosis. [12] This is consistent with our study where we found tuberculosis to be the culprit in 63.3% of the empyema patients.

In countries where tuberculosis is endemic, it is very important to differentiate between the two most common causes of exudative pleural effusion that are malignancy and tuberculosis, as their treatment and prognosis vary. It is a common practice in resource limited settings to start empiric anti-tuberculous therapy (ATT) in all patients with empyema, if the effusion fails to respond to regimen only then alternate diagnosis is considered. However, if malignant pleural effusion (MPE) is treated as tuberculosis it would not only become the source of unnecessary delay but also affects them with the adverse reaction of ATT [13]. YU Xiang et.al in their study conducted on 430 cases of tuberculous pleural effusion, confirmed that medical thoracoscopy is a safe and efficacious way of managing multiloculted and organized tuberculous pleural effusion [14]. It is reported in literature that in 26 to 54% of cases pleural thickening of 10 mm or more can cause symptoms in patients with tuberculous empyema [15]. So it is vital to re-expand the trapped lung and decrease residual pleural thickening.

Many terms have been used for rigid medical Thoracoscopy in literature, like video-assisted Thoracoscopy medical (VAT-M), non-intubated VATS, pleuroscopy (local anesthesia) or simply medical thoracoscopy. We have used the term medical Thoracoscopy in our article to avoid confusion with VATS as there is much debate between pulmonologists and thoracic surgeons on this subject.

One noteworthy feature of the approach to empyema is the liaison between pulmonologists and thoracic surgeons. These two specialties ought to share patients with clinical quandaries as pulmonologists need the surgical back-up and likewise thoracic surgeons would benefit from the availability of a keen clinician.

## Conclusion

Medical thoracoscopy in early management of multiloculated pleural empyema is perhaps a valuable intervention in underdeveloped countries where facilities of thoracic surgery are scant. All lymphocytic predominant pleural effusions are not tuberculosis. Medical thoracoscopy is also beneficial in timely diagnosis of tuberculous empyema and discriminating it from pyogenic empyema in countries with high burden of tuberculosis.

This is case series on management of empyema by medical thoracoscopy and further prospective studies are needed on the long term follow up of the cases.

## Abbreviations

ATT: Anti-tuberculous therapy
CT scan: Computed tomography scan
Fr: French unit
MHz: Mega hertz
MPE: Malignant pleural effusion
SPSS: Statistical analysis software package
VATS: Video-assissted thoracoscopic surgery
